# Supplementary material for: mTORC1 cooperates with tRNA wobble modification to sustain the protein synthesis machinery
Source: Nat Commun. 2025 May 6;16:4201. doi: 10.1038/s41467-025-59185-4 (PMC12056009; doi:10.1038/s41467-025-59185-4)
Supplement: Supplementary file 1 — Supplementary Information [file 41467_2025_59185_MOESM1_ESM.pdf]

Supplementary Information:

mTORC1 cooperates with tRNA wobble modification to sustain the protein synthesis machinery

Julia Hermann[1,2], Toman Borteçen[2,3], Robert Kalis[4,5], Alexander Kowar[2,6], Catarina Pechincha[1,2], Vivien Vogt[4,5], Martin Schneider[7], Dominic Helm[7], Jeroen Krijgsveld[3], Fabricio Loayza-Puch[6], Johannes Zuber[4,8], Wilhelm Palm[1]*

[1] Division of Cell Signaling and Metabolism, German Cancer Research Center (DKFZ) and DKFZ-ZMBH Alliance, Heidelberg, Germany

[2] Faculty of Biosciences, University of Heidelberg, Heidelberg, Germany

[3] Division of Proteomics of Stem Cells and Cancer, German Cancer Research Center (DKFZ), Heidelberg, Germany

[4] Research Institute of Molecular Pathology (IMP), Vienna BioCenter (VBC), Vienna, Austria

[5] Vienna BioCenter PhD Program, Doctoral School of the University of Vienna and Medical University of Vienna, Vienna BioCenter (VBC), Vienna, Austria

[6] Translational Control and Metabolism Group, German Cancer Research Center (DKFZ), Heidelberg, Germany

[7] Proteomics Core Facility, German Cancer Research Center (DKFZ), Heidelberg, Germany

[8] Medical University of Vienna, Vienna BioCenter (VBC), Vienna, Austria

* Corresponding author: w.palm@dkfz-heidelberg.de

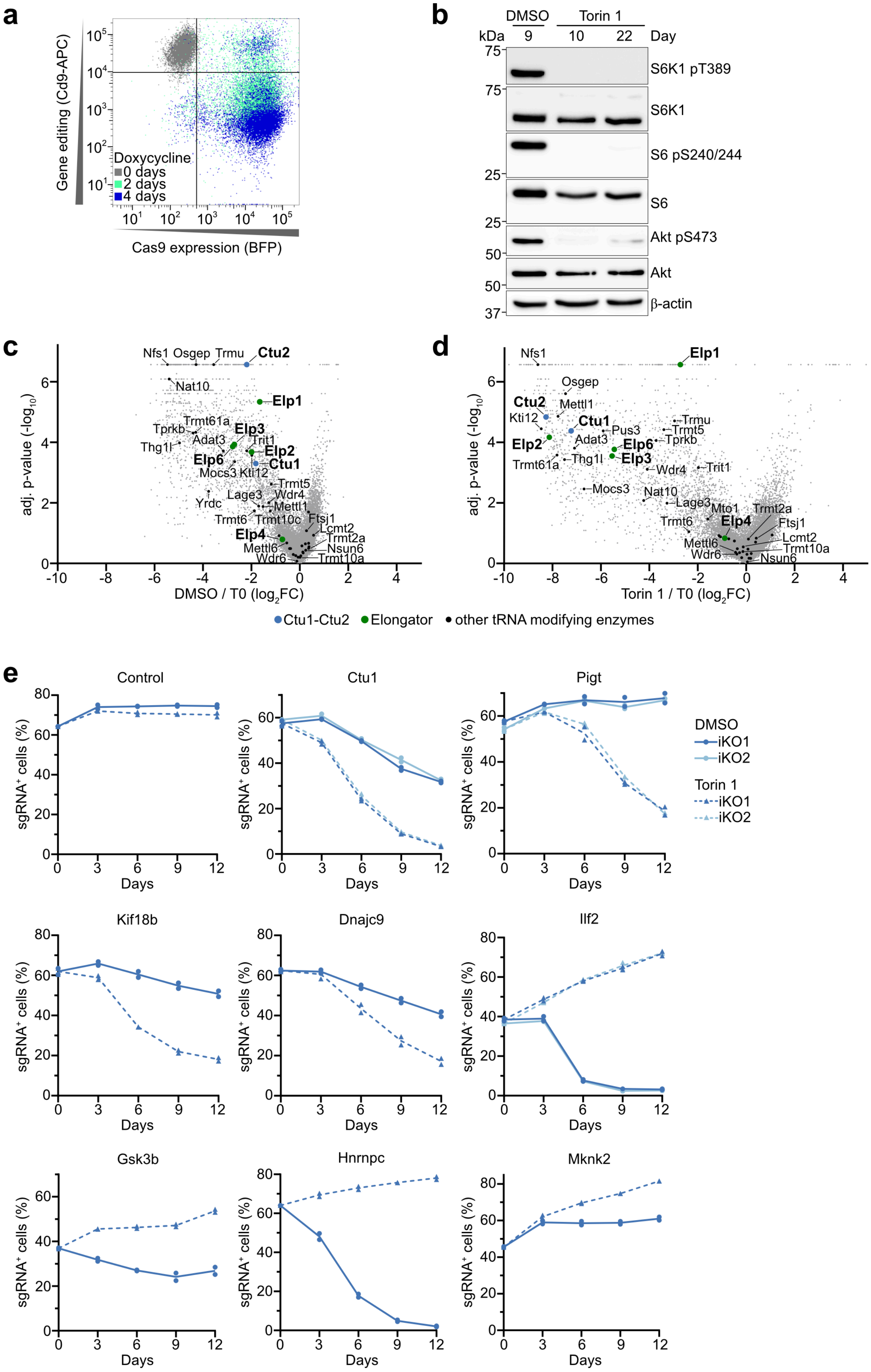

a
Gene editing (Cd9-APC)
Cas9 expression (BFP)
Doxycycline
0 days
2 days
4 days
b
DMSO
Torin 1
kDa
Day
9
10
22
S6K1 pT389
S6K1
S6 pS240/244
S6
Akt pS473
Akt
β-actin
c
adj. p-value (-log10)
DMSO / T0 (log2FC)
Nfs1
Osgep
Trmu
Ctu2
Nat10
Elp1
Trmt61a
Tprkb
Elp3
Adat3
Trit1
Thg1l
Elp2
Elp6
Ctu1
Mocs3
Kti12
Trmt5
Lage3
Wdr4
Yrdc
Mettl1
Trmt6
Trmt10c
Ftsj1
Lcmt2
Elp4
Trmt2a
Mettl6
Nsun6
Wdr6
Trmt10a
d
Torin 1 / T0 (log2FC)
Mettl1
Kti12
Pus3
Trmt5
Mocs3
Mto1
Ctu1-Ctu2
Elongator
other tRNA modifying enzymes
e
Control
Ctu1
Pigt
Kif18b
Dnajc9
Ilf2
Gsk3b
Hnrnpc
Mknk2
sgRNA+ cells (%)
Days
DMSO
iKO1
iKO2
Torin 1

Supplementary Figure 1 Cell proliferation-based CRISPR screen during mTOR inhibition

a) Analysis of Cas9-BFP expression and sgRNA-mediated gene editing of the surface protein Cd9 in EPP2 iCas9 (inducible Cas9) cells after addition of doxycycline [300 ng/ml] for the indicated periods, analyzed by flow cytometry (10,000 cells per condition). **b)** mTOR pathway activity in EPP2 iCas9 cells harvested at indicated time points during the CRISPR screen, analyzed by immunoblotting for phosphorylation targets of mTORC1 (S6K1 T389 and downstream S6 S240/244) and mTORC2 (Akt S473). **c)**, **d)** Gene-level enrichment or depletion of sgRNAs in the final cell populations of the CRISPR screen in c) DMSO and d) torin 1 [300 nM] compared to the starting population (T0). Selected hits are highlighted. **e)** Competitive proliferation assays of EPP2 iCas9 cells that express sgRNAs targeting indicated genes (iKO1/2) against non-sgRNA-expressing control cells ± torin 1 [300 nM]. The fraction of iKO cells (% $sgRNA^{+}$) is represented as mean (n = 2 technical replicates). Source data are provided as a Source Data file.

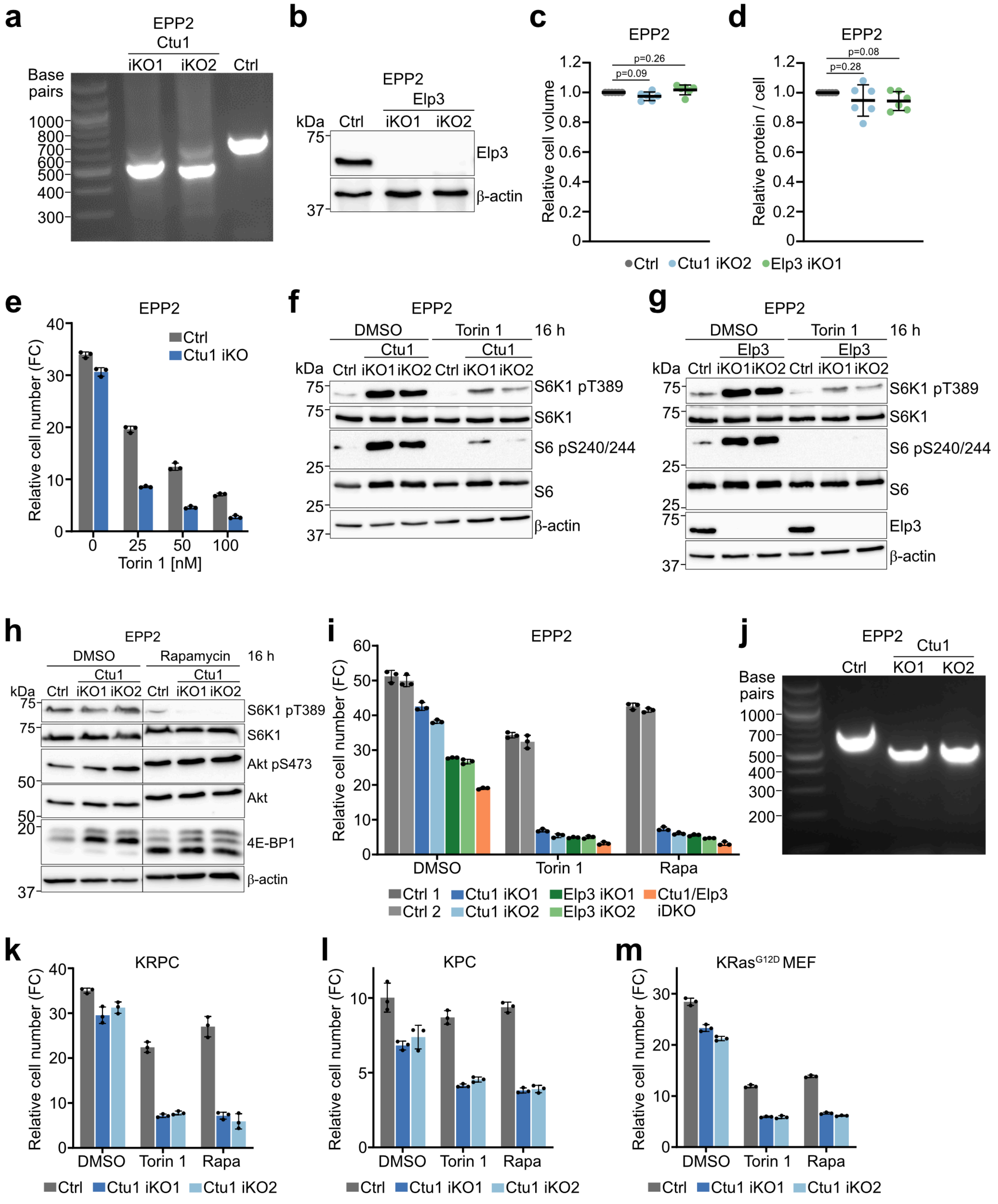
a
EPP2
Ctu1
iKO1 iKO2 Ctrl
Base pairs
1000
800
700
600
500
400
300
b
EPP2
Elp3
kDa Ctrl iKO1 iKO2
75
37
Elp3
β-actin
c
EPP2
p=0.26
p=0.09
Relative cell volume
1.2
1.0
0.8
0.6
0.4
0.2
0
d
EPP2
p=0.08
p=0.28
Relative protein / cell
1.2
1.0
0.8
0.6
0.4
0.2
0
Ctrl Ctu1 iKO2 Elp3 iKO1
e
EPP2
Ctrl
Ctu1 iKO
Relative cell number (FC)
40
30
20
10
0
0 25 50 100
Torin 1 [nM]
f
EPP2
DMSO Torin 1 16 h
Ctu1 Ctu1
kDa Ctrl iKO1 iKO2 Ctrl iKO1 iKO2
75
75
25
25
37
S6K1 pT389
S6K1
S6 pS240/244
S6
β-actin
g
EPP2
DMSO Torin 1 16 h
Elp3 Elp3
kDa Ctrl iKO1 iKO2 Ctrl iKO1 iKO2
75
75
25
25
75
37
S6K1 pT389
S6K1
S6 pS240/244
S6
Elp3
β-actin
h
EPP2
DMSO Rapamycin 16 h
Ctu1 Ctu1
kDa Ctrl iKO1 iKO2 Ctrl iKO1 iKO2
75
75
50
50
20
37
S6K1 pT389
S6K1
Akt pS473
Akt
4E-BP1
β-actin
i
EPP2
Relative cell number (FC)
60
50
40
30
20
10
0
DMSO Torin 1 Rapa
Ctrl 1 Ctu1 iKO1 Elp3 iKO1 Ctu1/Elp3 iDKO
Ctrl 2 Ctu1 iKO2 Elp3 iKO2
j
EPP2
Ctu1
Ctrl KO1 KO2
Base pairs
1000
700
500
400
300
200
k
KRPC
Relative cell number (FC)
40
30
20
10
0
DMSO Torin 1 Rapa
Ctrl Ctu1 iKO1 Ctu1 iKO2
l
KPC
Relative cell number (FC)
10
5
0
DMSO Torin 1 Rapa
Ctrl Ctu1 iKO1 Ctu1 iKO2
m
KRas G12D MEF
Relative cell number (FC)
30
20
10
0
DMSO Torin 1 Rapa
Ctrl Ctu1 iKO1 Ctu1 iKO2

Supplementary Figure 2 Ctu1 is required for cell proliferation during mTORC1 inhibition

a) Editing of *Ctu1* in Ctu1 iKO EPP2 cells after Cas9 induction for 4 days with doxycycline [300 ng/ml]. Successful editing was confirmed by PCR using primers that amplify the genomic *Ctu1* locus spanning the two sgRNA cutting sites. **b)** Loss of Elp3 in Elp3 iKO EPP2 cells after Cas9 induction for 4 days with doxycycline [300 ng/ml], analyzed by immunoblotting. **c)** Cell volume and **d)** protein/cell in Ctu1 iKO and Elp3 iKO EPP2 cells. Data are represented as replicate mean ± SD (n = 6 independent experiments with 3 technical replicates); p values were calculated by unpaired two-tailed *t*-test with Welch correction. **e)** Fold change (FC) in cell number of Ctu1 iKO EPP2 cells after 3 days in culture + torin 1 [0 – 100 nM]. Data are represented as mean ± SD (n = 3 technical replicates). **f)**, **g)** mTOR pathway activity in f) Ctu1 iKO and g) Elp3 iKO EPP2 cells after 16 h ± torin 1 [25 nM] , analyzed by immunoblotting. **h)** mTOR pathway activity in Ctu1 iKO EPP2 cells after 16 h ± rapamycin [12.5 nM], analyzed by immunoblotting. **i)** Fold change in cell number of Ctu1 iKO, Elp3 iKO and Ctu1/Elp3 iDKO EPP2 cells after 3 days in culture ± torin 1 [25 nM] or rapamycin [12.5 nM]. Data are represented as mean ± SD (n = 3 technical replicates). **j)** Editing of *Ctu1* in single cell-derived Ctu1 KO EPP2 cell lines, analyzed by PCR of the genomic *Ctu1* locus. PCR primers were as in a). **k)** – **m)** Fold change in cell number of Ctu1 iKO k) KRPC cells, l) KPC cells and m) $KRas^{G12D}$ MEFs after 3 days in culture ± torin 1 [25 nM] or rapamycin [12.5 nM]. Data are represented as mean ± SD (n = 3 technical replicates). Source data are provided as a Source Data file.

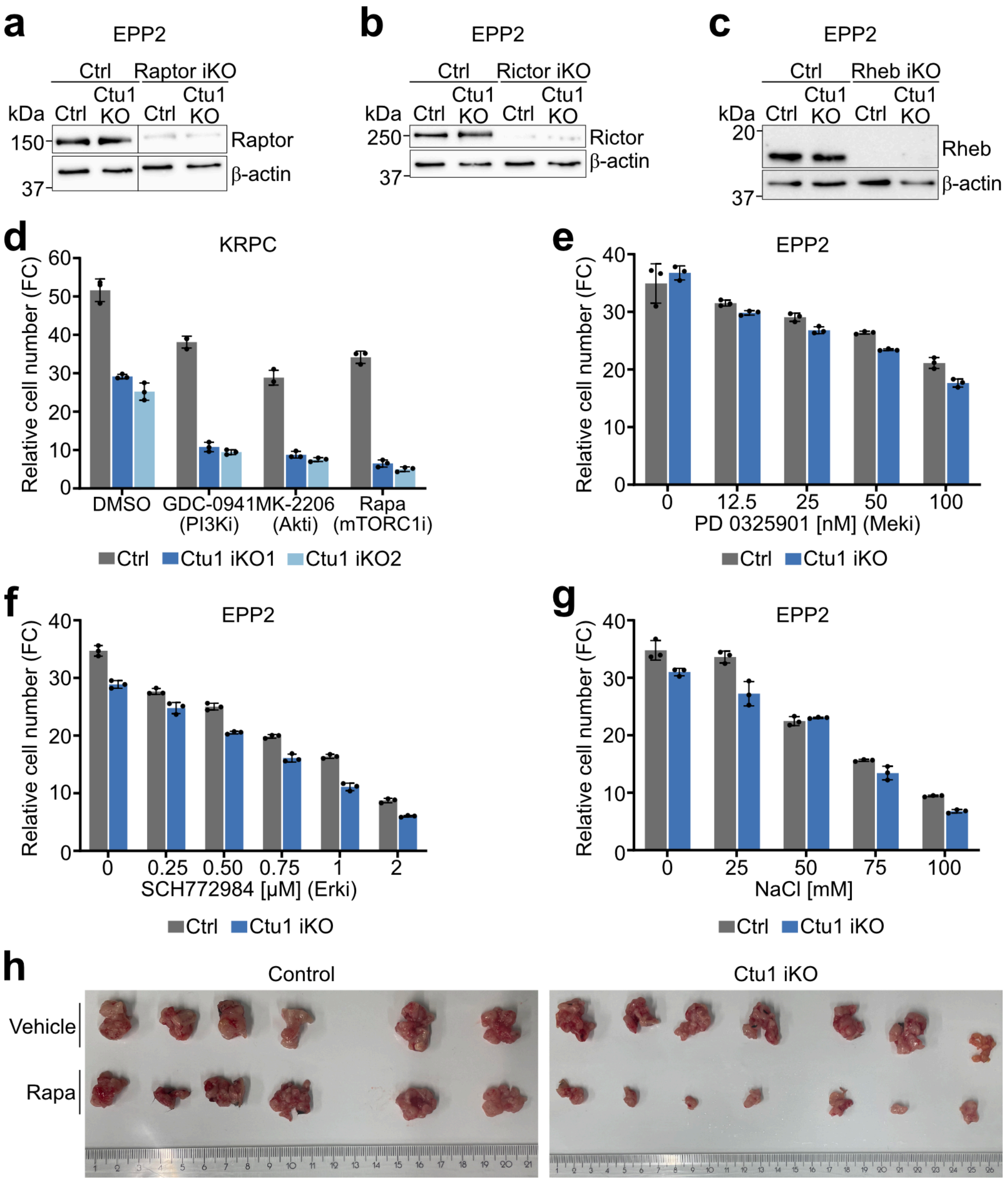

Supplementary Figure 3 Ctu1-deficient cells are hypersensitive to inhibition of the PI3-kinase-Akt-mTORC1 signaling pathway but not to general growth perturbations

a) – c) Loss of a) Raptor, b) Rictor and c) Rheb in Ctu1 KO and control EPP2 cells expressing sgRNAs against the respective gene after Cas9 induction for 4 days with doxycycline [300 ng/ml], analyzed by immunoblotting. **d)** Fold change in cell number of Ctu1 iKO KRPC cells after 3 days in culture ± GDC-0941 [1 µM], MK-2206 [2 µM] or rapamycin [12.5 nM]. Data are represented as mean ± SD (n = 3 technical replicates). **e) – g)** Fold change in cell number of Ctu1 iKO EPP2 cells after 3 days in culture + e) PD0325901 [0 – 100 nM], f) SCH772984 [0 – 2 µM] or g) NaCl [0 – 100 mM]. Data are represented as mean ± SD (n = 3 technical replicates). **h)** Images of orthotopic pancreatic tumors from Ctu1 iKO and control EPP2 cells in C57BL/6J Rag2$^{-/-}$ mice after 9 days treatment with rapamycin [5 mg/kg/day] or vehicle. Source data are provided as a Source Data file.

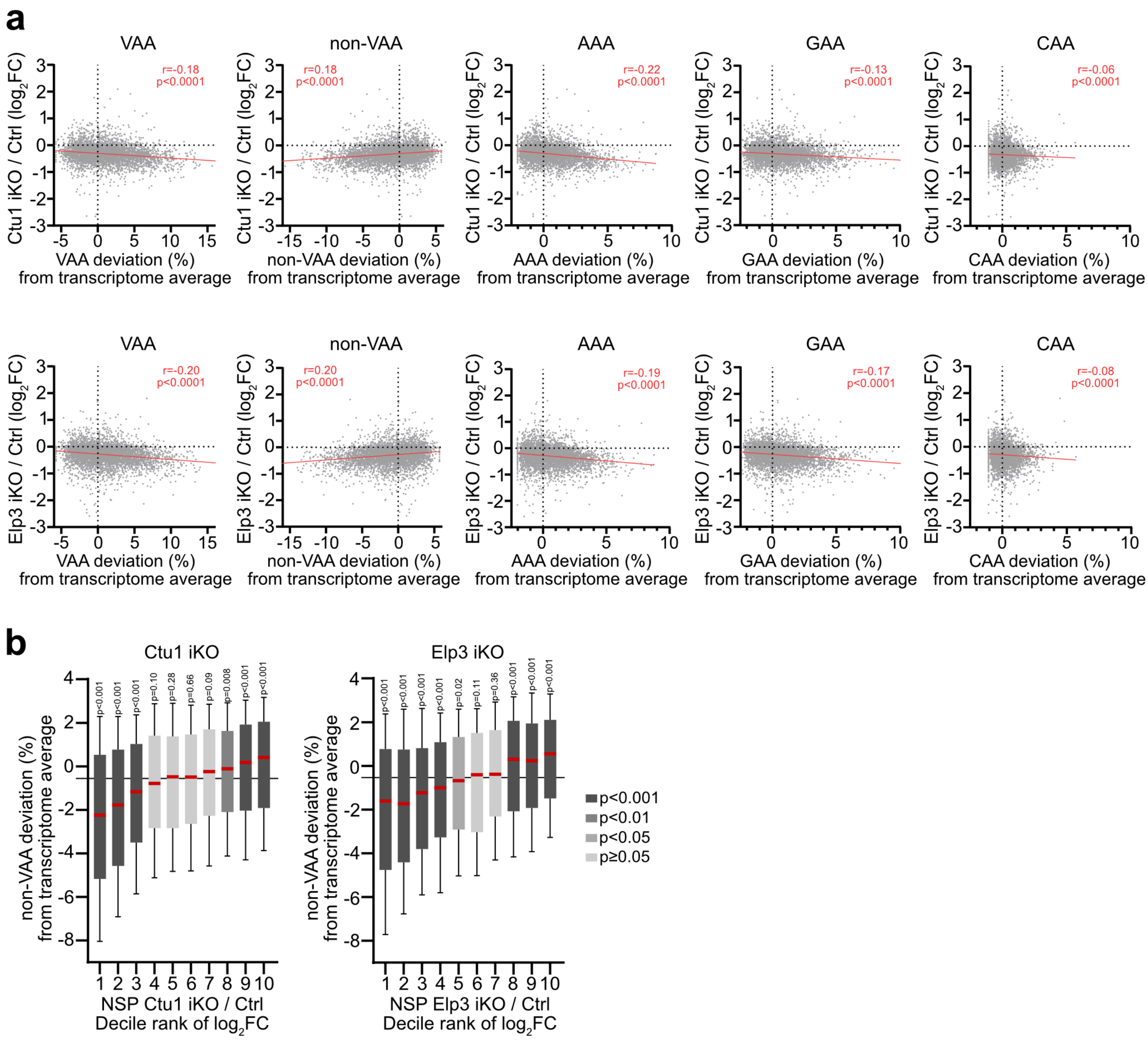

Supplementary Figure 4 Decreased protein synthesis and mRNA wobble codon content correlate in cells lacking tRNA wobble enzymes

a) Correlation plots comparing change of individual proteins in the newly synthesized proteome of Ctu1 iKO or Elp3 iKO EPP2 cells with their mRNA codon usage deviation (n = 3 experimental replicates). Shown are U_{34} wobble tRNA codons AAA, CAA, GAA (VAA codons) and non-VAA codons with Pearson correlation coefficient and two-tailed p value. **b)** Correlation of mRNA codon usage deviation and change in the newly synthesized proteome (NSP) of Ctu1 iKO or Elp3 iKO EPP2 cells for non-VAA codons (n = 3 experimental replicates). Newly synthesized proteome data are represented as decile ranks of log_2FC (i.e. 1 = 10 % most decreased proteins in iKO / Ctrl). In box plots, centre line represents median, upper and lower bounds of the box 75th and 25th percentiles, whiskers 10th to 90th percentiles; p values were calculated by two-tailed Wilcoxon rank test. Grey line represents median codon usage of all quantified proteins. Source data are provided as a Source Data file.

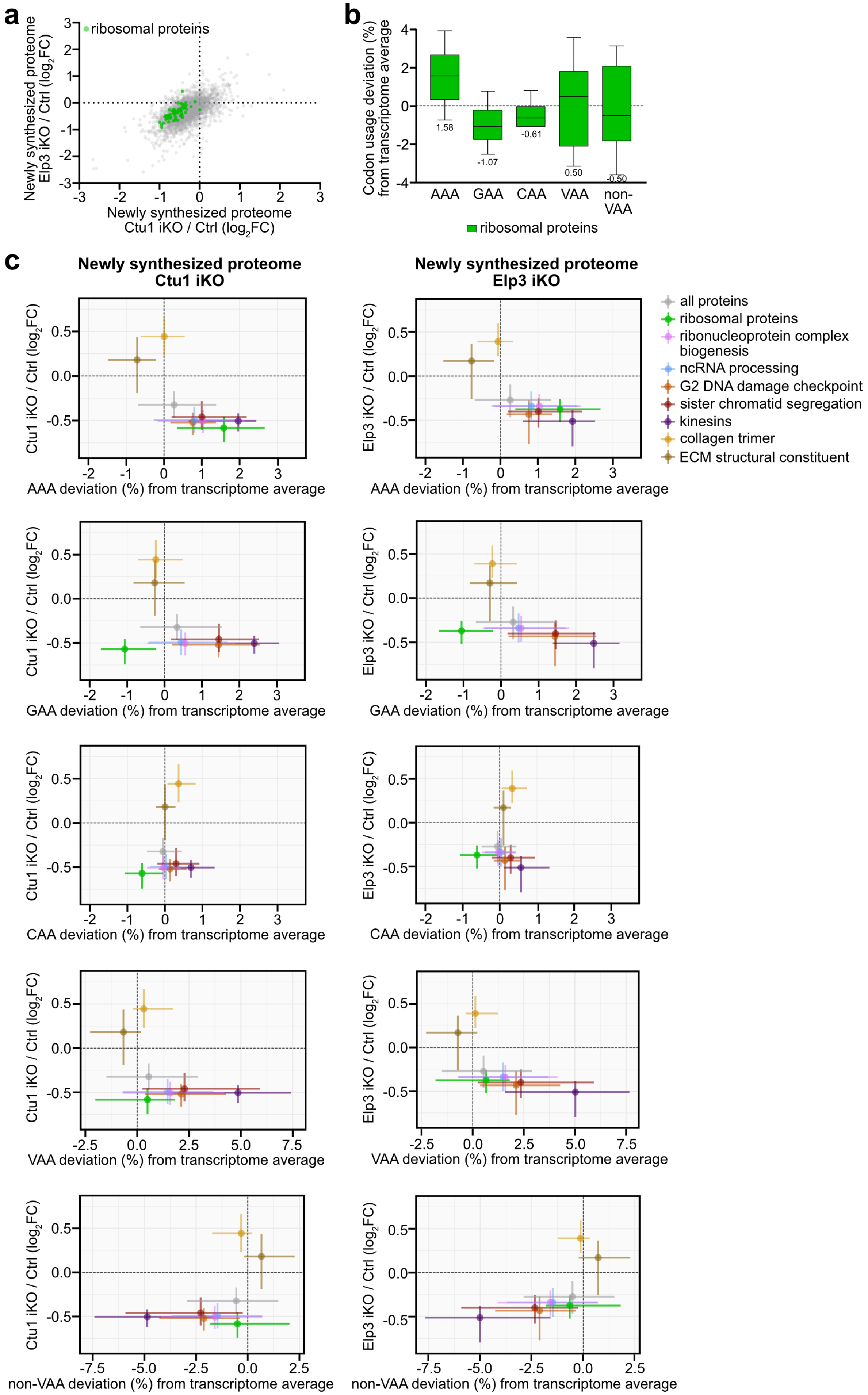

a
ribosomal proteins
Newly synthesized proteome Elp3 iKO / Ctrl (log_2FC)
Newly synthesized proteome Ctu1 iKO / Ctrl (log_2FC)
b
Codon usage deviation (%) from transcriptome average
1.58
-1.07
-0.61
0.50
-0.50
AAA
GAA
CAA
VAA
non-VAA
ribosomal proteins
c
Newly synthesized proteome Ctu1 iKO
Newly synthesized proteome Elp3 iKO
all proteins
ribosomal proteins
ribonucleoprotein complex biogenesis
ncRNA processing
G2 DNA damage checkpoint
sister chromatid segregation
kinesins
collagen trimer
ECM structural constituent
Ctu1 iKO / Ctrl (log_2FC)
Elp3 iKO / Ctrl (log_2FC)
AAA deviation (%) from transcriptome average
GAA deviation (%) from transcriptome average
CAA deviation (%) from transcriptome average
VAA deviation (%) from transcriptome average
non-VAA deviation (%) from transcriptome average

Supplementary Figure 5 Correlation between changes in protein synthesis and mRNA wobble codon content in cells deficient for Ctu1 or Elp3

a) Scatter plot comparing changes in the newly synthesized proteome of Ctu1 iKO and Elp3 iKO EPP2 cells. Ribosomal proteins are highlighted (n = 3 experimental replicates). **b)** mRNA codon usage deviation of ribosomal proteins that were quantified in the newly synthesized proteome of Ctu1 iKO EPP2 cells. In box plots, centre line represents median, upper and lower bounds of the box 75th and 25th percentiles, whiskers 10th to 90th percentiles. **c)** Correlation plots comparing changes in the newly synthesized proteome of Ctu1 iKO or Elp3 iKO EPP2 cells with mRNA codon usage deviation (n = 3 experimental replicates). Shown are protein groups whose synthesis is particularly decreased or increased in Ctu1-deficient cells. Data are represented as median and interquartile range. In b), c), analyzed are U_{34} wobble tRNA codons AAA, CAA, GAA (VAA codons) and non-VAA codons. Source data are provided as a Source Data file.

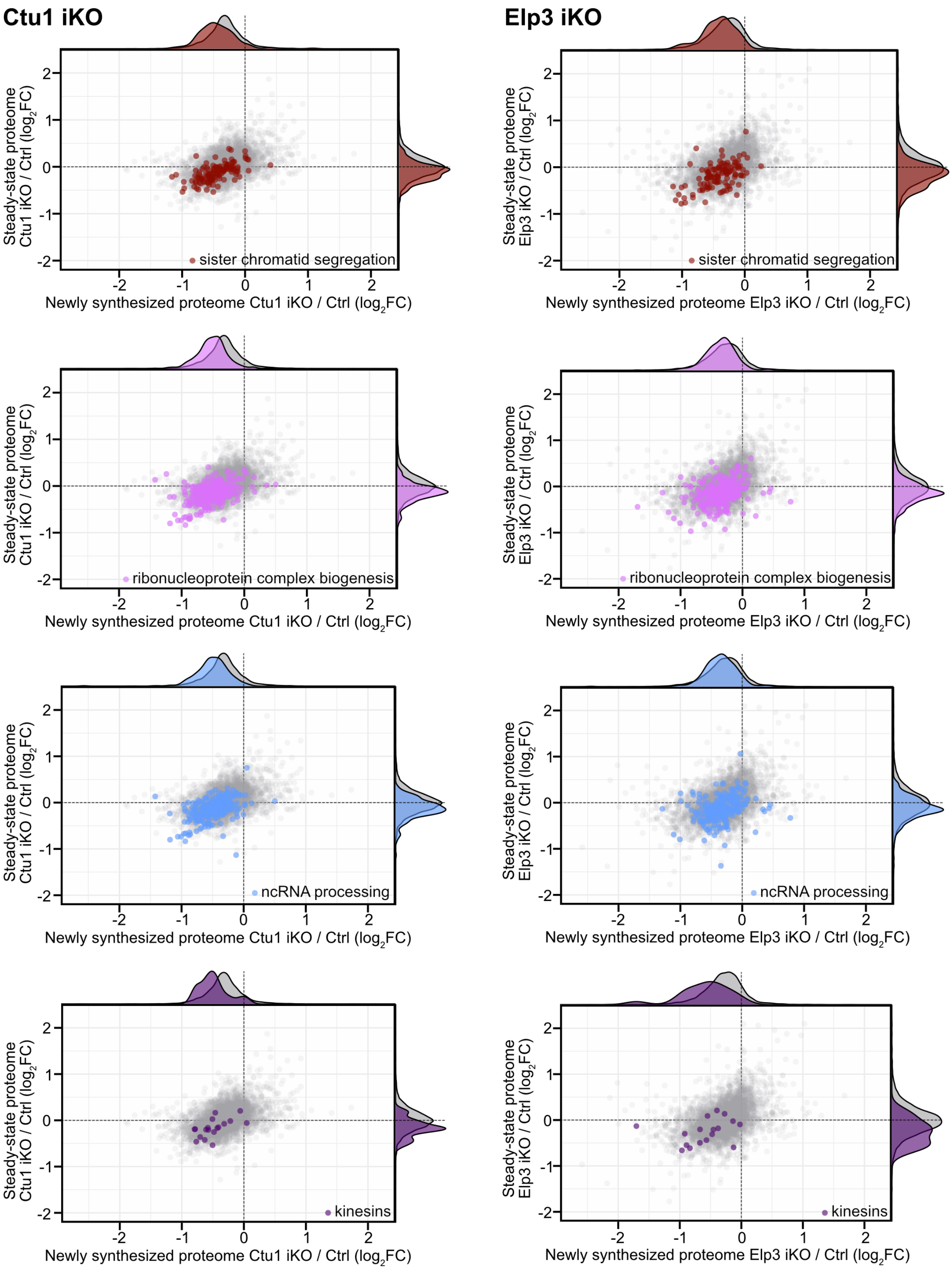

Supplementary Figure 6 Correlation between protein synthesis and protein abundance in cells deficient for Ctu1 or Elp3

Scatter plots comparing changes in the steady-state proteome (n = 5 independent experiments) versus newly synthesized proteome (n = 3 experimental replicates) of Ctu1 iKO or Elp3 iKO EPP2 cells. Selected protein groups whose synthesis is particularly dependent on U_{34}-enzymes are highlighted.

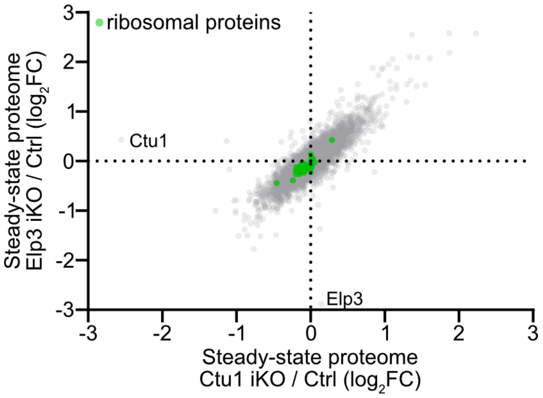

Supplementary Figure 7 Loss of Ctu1 or Elp3 leads to similar proteome changes

Scatter plot comparing changes in the steady-state proteome of Ctu1 iKO and Elp3 iKO EPP2 cells (n = 5 independent experiments). Source data are provided as a Source Data file.

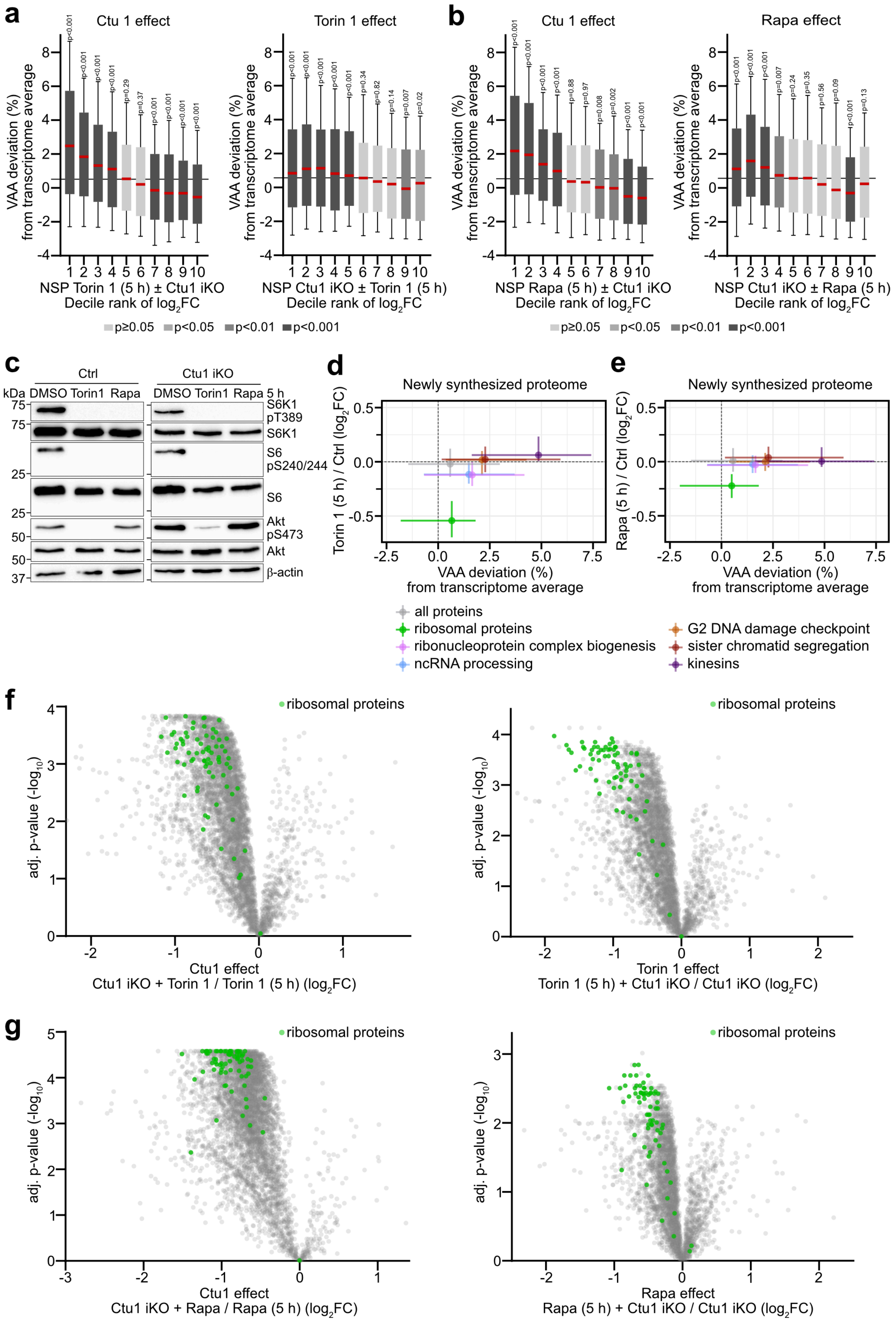
a
Ctu 1 effect
Torin 1 effect
VAA deviation (%) from transcriptome average
NSP Torin 1 (5 h) ± Ctu1 iKO
Decile rank of log2FC
NSP Ctu1 iKO ± Torin 1 (5 h)
Decile rank of log2FC
b
Ctu 1 effect
Rapa effect
NSP Rapa (5 h) ± Ctu1 iKO
Decile rank of log2FC
NSP Ctu1 iKO ± Rapa (5 h)
Decile rank of log2FC
p≥0.05 p<0.05 p<0.01 p<0.001
c
Ctrl
Ctu1 iKO
kDa DMSO Torin1 Rapa DMSO Torin1 Rapa 5 h
S6K1 pT389
S6K1
S6 pS240/244
S6
Akt pS473
Akt
β-actin
d
Newly synthesized proteome
Torin 1 (5 h) / Ctrl (log2FC)
VAA deviation (%) from transcriptome average
e
Newly synthesized proteome
Rapa (5 h) / Ctrl (log2FC)
VAA deviation (%) from transcriptome average
all proteins
ribosomal proteins
ribonucleoprotein complex biogenesis
ncRNA processing
G2 DNA damage checkpoint
sister chromatid segregation
kinesins
f
ribosomal proteins
adj. p-value (-log10)
Ctu1 effect
Ctu1 iKO + Torin 1 / Torin 1 (5 h) (log2FC)
Torin 1 effect
Torin 1 (5 h) + Ctu1 iKO / Ctu1 iKO (log2FC)
g
ribosomal proteins
Ctu1 effect
Ctu1 iKO + Rapa / Rapa (5 h) (log2FC)
Rapa effect
Rapa (5 h) + Ctu1 iKO / Ctu1 iKO (log2FC)

Supplementary Figure 8 Ribosomal protein synthesis is highly sensitive to concerted suppression of mTORC1 and Ctu1

a), **b)** Correlation of mRNA codon usage deviation and change in the newly synthesized proteome (NSP) of EPP2 cells after 5 h mTORC1 inhibitor ± Ctu1 iKO (Ctu1 effect) or Ctu1 iKO ± 5 h mTORC1 inhibitor (inhibitor effect). mTORC1 inhibitors were a) torin 1 [50 nM] and b) rapamycin [50 nM]. Newly synthesized proteome data are represented as decile ranks of log_2FC (i.e. 1 = 10 % most decreased proteins in iKO / Ctrl). In box plots, centre line represents median, upper and lower bounds of the box 75th and 25th percentiles, whiskers 10th to 90th percentiles; p values were calculated by two-tailed Wilcoxon rank test. Grey line represents median codon usage of all quantified proteins. **c)** mTOR pathway activity in Ctu1 iKO EPP2 cells after 5 h treatment ± torin 1 [50 nM] or rapamycin [50 nM], analyzed by immunoblotting. Cells were treated with SILAC-AHA medium and inhibitors as for the nascent proteomics experiments. **d)**, **e)** Correlation plot comparing mRNA VAA codon deviation versus change in the newly synthesized proteome of EPP2 cells after 5 h ± d) torin 1 [50 nM] or e) rapamycin [50 nM]. Shown are protein groups whose synthesis is particularly dependent on Ctu1. Data are represented as median and interquartile range. **f)**, **g)** Changes in the newly synthesized proteome of EPP2 cells showing Ctu1 iKO and mTORC1 inhibitor effects. mTORC1 inhibitors were f) torin 1 [50 nM] and g) rapamycin [50 nM]. Newly synthesized proteome n = 3 experimental replicates. Source data are provided as a Source Data file.

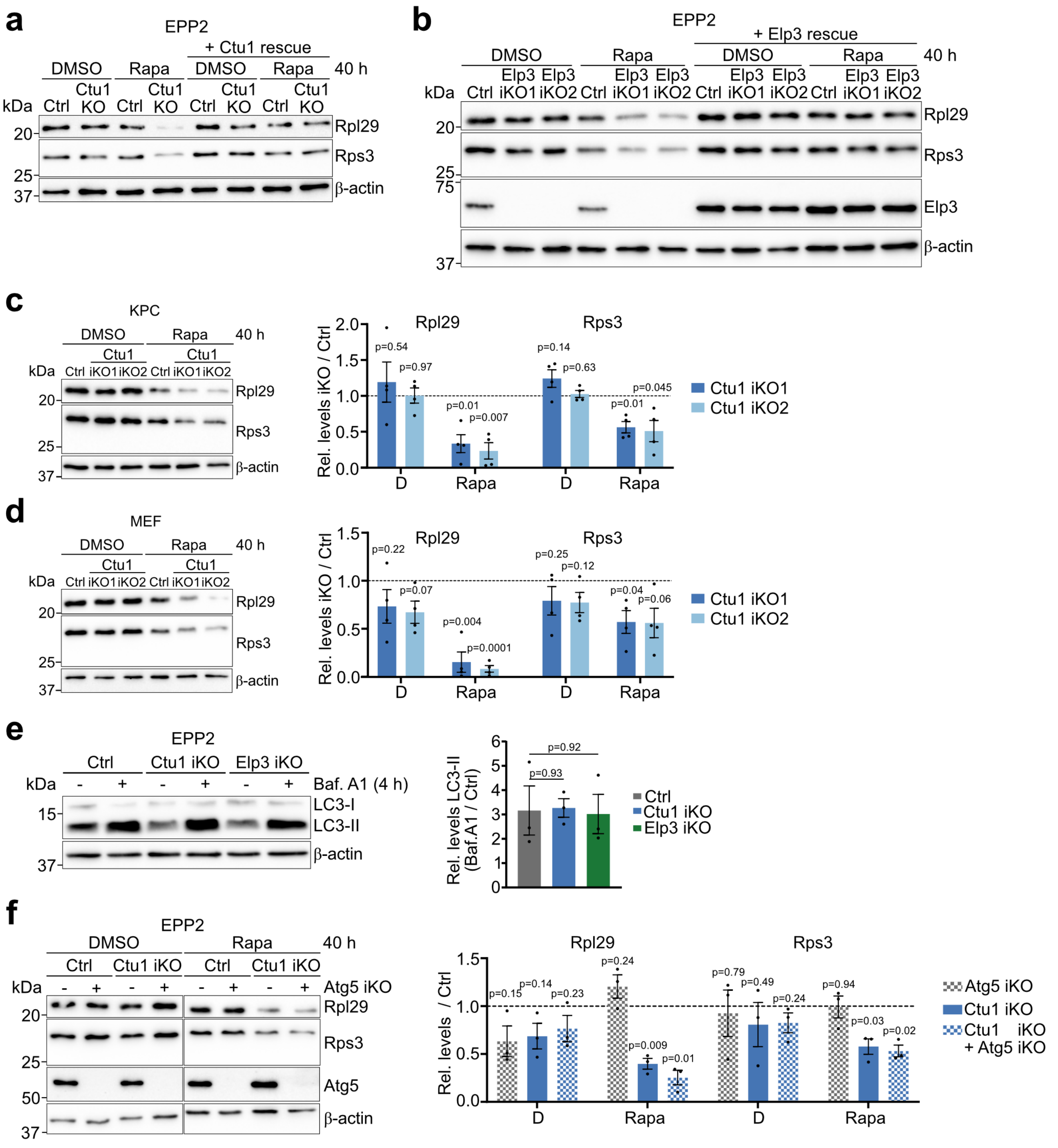

Supplementary Figure 9 mTORC1 and Ctu1 sustain the ribosomal protein levels independently of autophagy

a), b) Abundance of ribosomal proteins Rpl29 and Rps3 in a) Ctu1 KO and b) Elp3 iKO EPP2 cells with ectopic expression of Ctu1 / Elp3 cDNA after 40 h ± rapamycin [50 nM], analyzed by immunoblotting. **c), d)** Changes in Rpl29 and Rps3 abundance in Ctu1 iKO c) KPC cells and d) MEFs after 40 h ± rapamycin [50 nM], analyzed by quantitative immunoblotting. Data are normalized to sgRNA controls (dashed line) and represented as mean ± SEM (n = 4 independent experiments); p values were calculated by two-tailed one sample *t*-test with a hypothetical mean of 1. **e)** LC3-II flux

in Ctu1 iKO and Elp3 iKO EPP2 cells, analyzed by quantitative immunoblotting. Bafilomycin A1 treatment was 100 nM for 4 h. Data are normalized to DMSO control and represented as mean ± SEM (n = 3 independent experiments); p values were calculated by unpaired two-tailed *t*-test with Welch correction. **f)** Changes in Rpl29 and Rps3 abundance in Ctu1 / Atg5 iDKO EPP2 cells after 40 h ± rapamycin [50 nM], analyzed by quantitative immunoblotting. Data are normalized to sgRNA controls (dashed line) and represented as mean ± SEM (n = 3 independent experiments); p values were calculated by two-tailed one sample *t*-test with a hypothetical mean of 1. Source data are provided as a Source Data file.

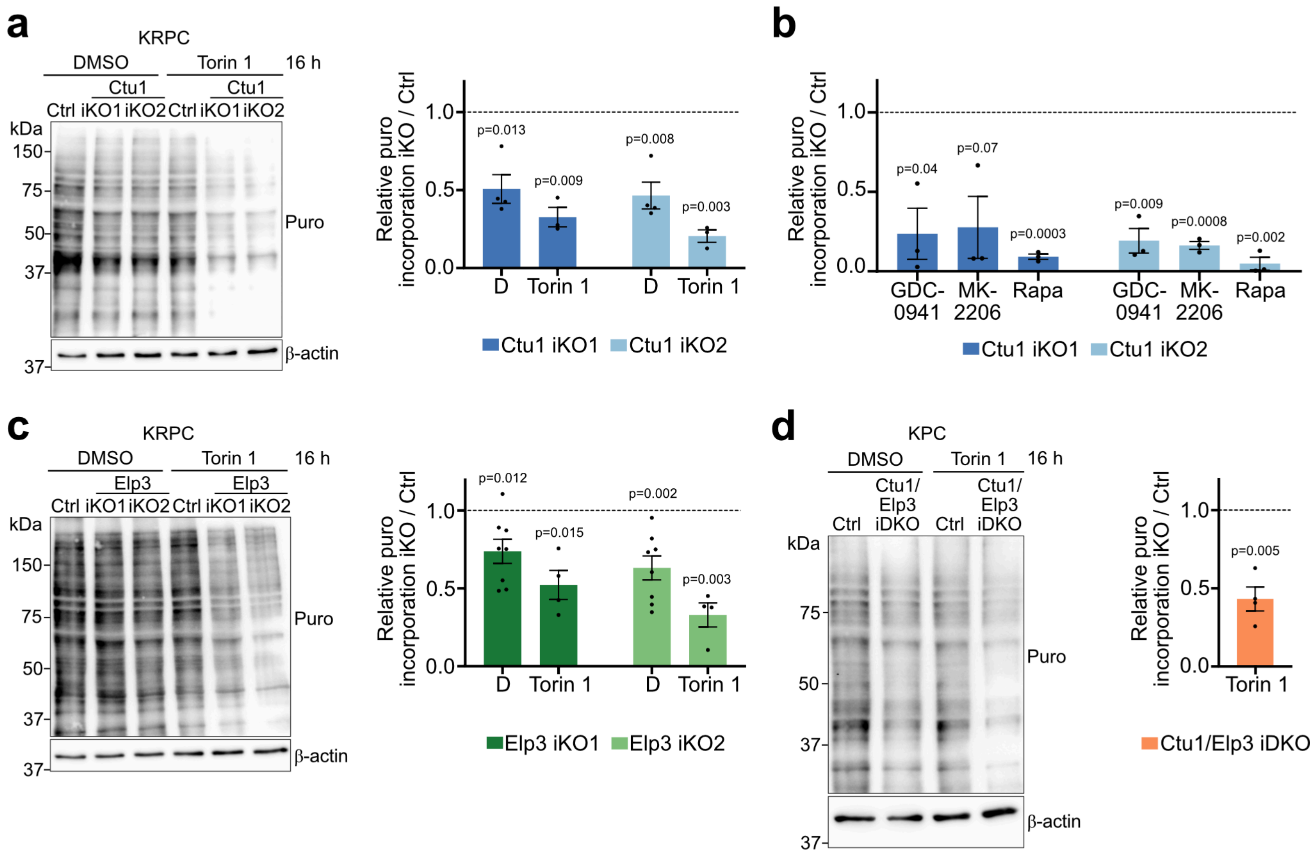

Supplementary Figure 10 Concerted suppression of mTORC1 and tRNA wobble enzymes inhibits protein synthesis

a) – d) Puromycin (puro) incorporation assay in U34-enzyme-deficient cells after 16 h inhibitor treatment, analyzed by quantitative immunoblotting. a) Ctu1 iKO KRPC cells ± torin 1 [300 nM]; b) Ctu1 iKO KRPC cells ± GDC-0941 [1 µM], MK-2206 [2 µM], rapamycin [12.5 nM]; c) Elp3 iKO KRPC cells ± torin 1 [300 nM]; d) Ctu1/Elp3 iDKO KPC cells ± torin 1 [300 nM]. Data are normalized to sgRNA controls (dashed line) and represented as mean ± SEM (a) DMSO n = 4, torin 1 n = 3, b) n = 3, c) DMSO n = 8, torin 1 n = 4, d) n = 4 independent experiments); p values were calculated by two-tailed one sample *t*-test with a hypothetical mean of 1. Source data are provided as a Source Data file.
