# Supplementary material for: mTORC1 cooperates with tRNA wobble modification to sustain the protein synthesis machinery
Source: Nat Commun. 2025 May 6;16:4201. doi: 10.1038/s41467-025-59185-4 (PMC12056009; doi:10.1038/s41467-025-59185-4)
Supplement: Supplementary file 2 — Description of Additional Supplementary Information [file 41467_2025_59185_MOESM2_ESM.pdf]

Description of Additional Supplementary Files

File Name: Supplementary Data 1
Description: *sgRNA counts from CRISPR screens.* Raw sgRNA counts from CRISPR screens in EPP2 cells to identify genes that become essential during mTOR inhibition. For each screening condition, raw sequencing reads of individual sgRNA sequences are provided. Non-targeting genes indicate control sgRNAs. Column names describe condition_replicate (for T0) or the respective condition (DMSO, torin 1 [300 nM]).

File Name: Supplementary Data 2
Description: *MAGeCK analysis of CRISPR screens.* MAGeCK analysis of CRISPR screens in EPP2 cells. The table shows datasets for each condition (DMSO, torin 1 [300 nM]) compared to T0 and for endpoint comparison (torin 1 versus DMSO). Mediannormalized log2 fold-changes, p-values and FDRs were calculated by MAGeCK v0.5.9.

File Name: Supplementary Data 3
Description: *Nascent proteomics datasets for Ctu1 iKO, Elp3 iKO and mTORC1 inhibitors.* Nascent proteomics datasets for SILAC-AHA-enriched proteins from EPP2 cells. Samples were generated in 3 experimental replicates. Log2 fold-changes and spectral count (adjusted) p values (p value of DEqMS considering the number of detected peptides) for Ctu1 iKO versus control, Elp3 iKO versus control, mTORC1 inhibitor versus DMSO, Ctu1 iKO + mTORC1 inhibitor versus Ctu1 iKO + DMSO (inhibitor effect), and Ctu1 iKO + mTORC1 inhibitor versus mTORC1 inhibitor (Ctu1 effect) are provided. mTORC1 inhibitors were 5 h torin 1 [50 nM] or rapamycin [50 nM]. n = 3 experimental replicates.

File Name: Supplementary Data 4
Description: *RNA sequencing datasets for Ctu1 iKO.* and Elp3 iKO RNA sequencing of total RNA from EPP2 cells. Samples were generated in 2 biological replicates. Log2 fold-changes and (adjusted) p-values for Ctu1 iKO versus control and Elp3 iKO versus control are provided. n = 2 independent experiments.

File Name: Supplementary Data 5
Description: *Codon usage and nascent proteomics data of U34- enzyme-dependent transcripts.* Murine mRNA codon usage (% deviation from transcriptome average) for AAA, GAA, CAA, VAA and non-VAA codons and nascent proteomics data (log2 foldchange, adjusted p-value) for nascent proteomics datasets of Ctu1 iKO versus control and Elp3 iKO versus control (as in Suppl. Data 3); shown are the U34-enzymedependent protein groups as in Suppl. Fig. 5c.

File Name: Supplementary Data 6
Description: *Steady-state proteomics datasets for Ctu1 iKO, Elp3 iKO and rapamycin.* Steady-state proteomics of whole-cell lysates from EPP2 cells. Samples were generated in 5 biological replicates. Log2 fold-changes and (adjusted) p-values for Ctu1 iKO versus control, Elp3 iKO versus control, rapamycin versus DMSO, Ctu1 iKO + rapamycin versus control + DMSO or Elp3 iKO + rapamycin versus control + DMSO are provided. 40 h rapamycin [50 nM]; n = 5 independent experiments.

File Name: Supplementary Data 7
Description: *Transcriptome-wide usage of U34-enzyme-dependent codons.* Murine mRNA codon usage (% deviation from transcriptome average) for transcripts identified in nascent proteomics experiments are provided for AAA, GAA, CAA, VAA and non-VAA codons.

File Name: Supplementary Data 8
Description: sgRNA sequences used in this paper.
