# Supplementary material for: mTORC1 cooperates with tRNA wobble modification to sustain the protein synthesis machinery
Source: Nat Commun. 2025 May 6;16:4201. doi: 10.1038/s41467-025-59185-4 (PMC12056009; doi:10.1038/s41467-025-59185-4)
Supplement: Supplementary file 11 — Reporting Summary [file 41467_2025_59185_MOESM11_ESM.pdf]

nature portfolio

Corresponding author(s): Wilhelm Palm

Last updated by author(s): Apr 7, 2025

Reporting Summary

Nature Portfolio wishes to improve the reproducibility of the work that we publish. This form provides structure for consistency and transparency in reporting. For further information on Nature Portfolio policies, see our Editorial Policies and the Editorial Policy Checklist.

Statistics

For all statistical analyses, confirm that the following items are present in the figure legend, table legend, main text, or Methods section.

n/a	Confirmed	
☐	☒	The exact sample size (*n*) for each experimental group/condition, given as a discrete number and unit of measurement
☐	☒	A statement on whether measurements were taken from distinct samples or whether the same sample was measured repeatedly
☐	☒	The statistical test(s) used AND whether they are one- or two-sided<br>*Only common tests should be described solely by name; describe more complex techniques in the Methods section.*
☒	☐	A description of all covariates tested
☐	☒	A description of any assumptions or corrections, such as tests of normality and adjustment for multiple comparisons
☐	☒	A full description of the statistical parameters including central tendency (e.g. means) or other basic estimates (e.g. regression coefficient) AND variation (e.g. standard deviation) or associated estimates of uncertainty (e.g. confidence intervals)
☐	☒	For null hypothesis testing, the test statistic (e.g. *F*, *t*, *r*) with confidence intervals, effect sizes, degrees of freedom and *P* value noted<br>*Give P values as exact values whenever suitable.*
☒	☐	For Bayesian analysis, information on the choice of priors and Markov chain Monte Carlo settings
☒	☐	For hierarchical and complex designs, identification of the appropriate level for tests and full reporting of outcomes
☒	☐	Estimates of effect sizes (e.g. Cohen's *d*, Pearson's *r*), indicating how they were calculated

Our web collection on statistics for biologists contains articles on many of the points above.

Software and code

Policy information about availability of computer code

Data collection	FACS: cells were sorted using BD FACSDiva software (v8.0, FACSAriaIII); immunoblotting: ChemiDoc Touch imaging system (BioRad); proteomics: Orbitrap Tune (2.11 QF2 Build 30007) and XCalibur (4.4) software from Thermo Fischer Scientific.
Data analysis	Image Lab software (BioRad v6.0.0.25), GraphPad Prism (v10.1.0(316)), Bowtie 2 (v2.3.0), fastx-toolkit (v0.0.14), featureCounts (v1.6.1), MAGeCK (v0.5.9), Spectronaut (Biognosys, version 17.1.221229.55965), DIA-NN (version 1.8.1), R software environment (version 4.0.3), Limma (version 3.46.0), DEqMS (version 1.8.0), clusterProfiler (version 3.18.0), cutadapt (v3.4), FASTX-toolkit (v0.0.6), umi_tools (v1.1.1), STAR (v2.5.3a), DESeq2 (version 1.30.1). Custom scripts have been deposited as described in the methods section at https://github.com/ZuberLab/crispr-process-nf, https://github.com/ZuberLab/crispr-mageck-nf, https://github.com/DKFZ-ODCF/RNAseqWorkflow, https://github.com/A-X-Smitt/B250_diricore.

For manuscripts utilizing custom algorithms or software that are central to the research but not yet described in published literature, software must be made available to editors and reviewers. We strongly encourage code deposition in a community repository (e.g. GitHub). See the Nature Portfolio guidelines for submitting code & software for further information.

Data

Policy information about availability of data

All manuscripts must include a data availability statement. This statement should provide the following information, where applicable:

- Accession codes, unique identifiers, or web links for publicly available datasets
- A description of any restrictions on data availability
- For clinical datasets or third party data, please ensure that the statement adheres to our policy

CRISPR Screen, Proteomics and RNA Seq data are included in the Supplementary information files of the manuscript. The mass spectrometry data generated in this study have been deposited at ProteomeXchange via the PRIDE partner repository with identifiers PXD047316, PXD047935, the RiboSeq and RNA sequencing data at GEO via identifier GSE250593.

Research involving human participants, their data, or biological material

Policy information about studies with human participants or human data. See also policy information about sex, gender (identity/presentation), and sexual orientation and race, ethnicity and racism.

Reporting on sex and gender	n/a
Reporting on race, ethnicity, or other socially relevant groupings	n/a
Population characteristics	n/a
Recruitment	n/a
Ethics oversight	n/a

Note that full information on the approval of the study protocol must also be provided in the manuscript.

Field-specific reporting

Please select the one below that is the best fit for your research. If you are not sure, read the appropriate sections before making your selection.

☒ Life sciences ☐ Behavioural & social sciences ☐ Ecological, evolutionary & environmental sciences

For a reference copy of the document with all sections, see nature.com/documents/nr-reporting-summary-flat.pdf

Life sciences study design

All studies must disclose on these points even when the disclosure is negative.

Sample size	No statistical methods were used to estimate sample sizes. Sample sizes were chosen in accordance with standard practices. For animal experiments, sample size was determined based on previous studies conducted using similar setups, without the need for additional statistical estimations.
Data exclusions	For puromycin incorporation western blots, an unspecific band at around 42 kDa was excluded from quantification as this band appears only after long secondary antibody incubation, including in samples not treated with puromycin. For other experiments, no data were excluded.
Replication	The number of replicates in each experiment is specified in the figure legends. All results were successfully replicated in independent experiments at least twice, with comparable results.
Randomization	Samples for LC-MS analysis were acquired in a randomized manner. For other experiments, randomization was not relevant.
Blinding	The investigators were not blinded during data allocation and analysis.

Reporting for specific materials, systems and methods

We require information from authors about some types of materials, experimental systems and methods used in many studies. Here, indicate whether each material, system or method listed is relevant to your study. If you are not sure if a list item applies to your research, read the appropriate section before selecting a response.

Materials & experimental systems

n/a	Involved in the study	
	x	Antibodies
	x	Eukaryotic cell lines
x		Palaeontology and archaeology
	x	Animals and other organisms
x		Clinical data
x		Dual use research of concern
x		Plants

Methods

n/a	Involved in the study	
x		ChIP-seq
	x	Flow cytometry
x		MRI-based neuroimaging

Antibodies

Antibodies used	Antibodies were from Cell Signaling (#2920 Akt(pan), #4060 Phospho-Akt (Ser473), #2217 S6 Ribosomal Protein, #2215 Phospho-S6 Ribosomal Protein (Ser240/244), #2708 p70 S6 Kinase, #9234 Phospho-p70 S6 Kinase (Thr389), #5728 Elp3, #9538 Ribosomal Protein S3), ProteinTech (15799-1-AP Ribosomal Protein L29) and Sigma Aldrich (A5441 β-actin, MABE343 Puromycin). Secondary antibodies were from Sigma Aldrich (HRP anti-rabbit, HRP anti-mouse). Antibody for Flow Cytometry was from Invitrogen (17-0091-82 Cd9 Monoclonal Antibody KMC8, APC, eBioscience).
Validation	The Elp3 antibody (Cell Signaling 5728) was validated by CRISPR-mediated knockout followed by western blotting. The Cd9 antibody for Flow Cytometry (Invitrogen (17-0091-82 Cd9 Monoclonal Antibody KMC8, APC, eBioscience) was validated by CRISPR mediated knockout followed by flow cytometry analysis. Remaining antibodies (Cell Signaling (#2920 Akt(pan), #4060 Phospho-Akt (Ser473), #2217 S6 Ribosomal Protein, #2215 Phospho-S6 Ribosomal Protein (Ser240/244), #2708 p70 S6 Kinase, #9234 Phospho-p70 S6 Kinase (Thr389), #9538 Ribosomal Protein S3), ProteinTech (15799-1-AP Ribosomal Protein L29) and Sigma Aldrich (A5441 β-actin, MABE343 Puromycin)) were validated by the respective manufacturers.

Eukaryotic cell lines

Policy information about cell lines and Sex and Gender in Research

Cell line source(s)	EPP2, KRPC, SV40 large T antigen-immortalized MEFs and KPC (Pechincha et al., Science 2023), T24 (ATCC HTB-4), HEK 293T (ATCC CRL-3216)
Authentication	Human cell lines were authenticated by Single Nucleotide Polymorphism (SNP) typing by Multiplexion.
Mycoplasma contamination	All used cell lines were routinely tested and confirmed negative for mycoplasma contamination.
Commonly misidentified lines (See ICLAC register)	No commonly misidentified cell lines were used.

Animals and other research organisms

Policy information about studies involving animals; ARRIVE guidelines recommended for reporting animal research, and Sex and Gender in Research

Laboratory animals	16 and 24 week-old (mixed between groups) female and male (mixed between groups) C57BL/6J Rag2-/- mice
Wild animals	n/a
Reporting on sex	female and male (mixed between groups)
Field-collected samples	n/a
Ethics oversight	Animal experiments were performed in accordance with a protocol approved by the local Ministry (Research Institute of Molecular Pathology (IMP), Vienna BioCenter (VBC), Vienna, Austria).

Note that full information on the approval of the study protocol must also be provided in the manuscript.

Plants

Seed stocks: n/a

Novel plant genotypes: n/a

Authentication: n/a

Flow Cytometry

Plots

Confirm that:

- [x] The axis labels state the marker and fluorochrome used (e.g. CD4-FITC).
- [x] The axis scales are clearly visible. Include numbers along axes only for bottom left plot of group (a 'group' is an analysis of identical markers).
- [x] All plots are contour plots with outliers or pseudocolor plots.
- [x] A numerical value for number of cells or percentage (with statistics) is provided.

Methodology

Sample preparation: Resuspend cell pellet, incubate in blocking buffer (3 % BSA in PBS with 5 % FBS) for 30 min on ice protected from light. Spin down (100 g, 4 °C, 5 min), aspirate supernatant, resuspend cell pellet in 100 µl antibody solution (5 ug / mL in blocking buffer), incubate for 30 min on ice protected from light. Spin down (100 g, 4 °C, 5 min), aspirate supernatant, resuspend cell pellet in 500 µL washing buffer (PBS with 5 % FBS). Pass samples through cell strainer before measurement.

Instrument: BD FACS CantoII Flow Cytometry System

Software: BD FACSDiva software (v8.0) and FlowJo (v10.6.1)

Cell population abundance: 10,000 cells were measured for each experimental group.

Gating strategy: FSC-A vs SSC-A to select viable cells; SSC-A vs SSC-H to select single cells from viable cells; VL-450/50-A vs RL-670/14-A to determine Cas9 expression (VL450/50) and editing of Cd9 (RL-670/14); adjust PMT voltages with non-antibody treated sample.

- [ ] Tick this box to confirm that a figure exemplifying the gating strategy is provided in the Supplementary Information.
